# Transcriptional analyses of differential cultivars during resistant and susceptible interactions with *Peronospora effusa*, the causal agent of spinach downy mildew

**DOI:** 10.1038/s41598-020-63668-3

**Published:** 2020-04-21

**Authors:** Shyam L. Kandel, Amanda M. Hulse-Kemp, Kevin Stoffel, Steven T. Koike, Ainong Shi, Beiquan Mou, Allen Van Deynze, Steven J. Klosterman

**Affiliations:** 1 USDA-ARS, Crop Improvement and Protection Research Unit, Salinas, CA 93905 USA; 2 Department of Plant Sciences, University of California, Davis, CA, 95616 USA; 3 USDA-ARS, Genomics and Bioinformatics Research Unit, Raleigh, NC 27695 USA; 4 TriCal Diagnostics, Hollister, CA 95023 USA; 5 Department of Horticulture, University of Arkansas, Fayetteville, AR 72701 USA

**Keywords:** Molecular biology, Plant sciences

## Abstract

Downy mildew of spinach is caused by the obligate oomycete pathogen, *Peronospora effusa*. The disease causes significant economic losses, especially in the organic sector of the industry where the use of synthetic fungicides is not permitted for disease control. New pathotypes of this pathogen are increasingly reported which are capable of breaking resistance. In this study, we took advantage of new spinach genome resources to conduct RNA-seq analyses of transcriptomic changes in leaf tissue of resistant and susceptible spinach cultivars Solomon and Viroflay, respectively, at an early stage of pathogen establishment (48 hours post inoculation, hpi) to a late stage of symptom expression and pathogen sporulation (168 hpi). Fold change differences in gene expression were recorded between the two cultivars to identify candidate genes for resistance. In Solomon, the hypersensitive inducible genes such as pathogenesis-related gene PR-1, glutathione-S-transferase, phospholipid hydroperoxide glutathione peroxidase and peroxidase were significantly up-regulated uniquely at 48 hpi and genes involved in zinc finger CCCH protein, glycosyltransferase, 1-aminocyclopropane-1-carboxylate oxidase homologs, receptor-like protein kinases were expressed at 48 hpi through 168 hpi. The types of genes significantly up-regulated in Solomon in response to the pathogen suggests that salicylic acid and ethylene signaling pathways mediate resistance. Furthermore, many genes involved in the flavonoid and phenylpropanoid pathways were highly expressed in Viroflay compared to Solomon at 168 hpi. As anticipated, an abundance of significantly down-regulated genes was apparent at 168 hpi, reflecting symptom development and sporulation in cultivar Viroflay, but not at 48 hpi. In the pathogen, genes encoding RxLR-type effectors were expressed during early colonization of cultivar Viroflay while crinkler-type effector genes were expressed at the late stage of the colonization. Our results provide insights on gene expression in resistant and susceptible spinach-*P. effusa* interactions, which can guide future studies to assess candidate genes necessary for downy mildew resistance in spinach.

## Introduction

The demand for prepackaged ready to eat salad mixes has resulted in increased production of leafy greens in recent years. Currently, spinach is cultivated in more than 60 countries globally, with over 53 million tons of total production annually^1^. In the US, nearly four hundred thousand tons of spinach are produced every year^1^. There has also been a rapid increase in the demand for spinach in the US, beginning in the 1990s^2,3^. The Salinas Valley of California is sometimes referred to as ‘Salad Bowl of America’ since this region produces the majority of leafy greens grown in the US as well as nearly half of the total spinach in California^4,5^.

Downy mildew disease of spinach is caused by the obligate oomycete pathogen *Peronospora effusa* (Phylum Oomycota, Kingdom Stramenopila)^6^. Downy mildew on spinach can sometimes cause 100% yield loss in organic production systems, though the disease can be effectively managed by the applications of synthetic fungicides in conventional production. The use of resistant cultivars is the most promising control measure to minimize the downy mildew damage in organic spinach as well as to help reduce chemical usage for disease control in conventional spinach production. *P. effusa* is limited in its host range to spinach, and therefore spinach must be present for either sexual or asexual reproduction^7–9^. Given what is known about related species of *Peronospora*, the thick-walled, sexually produced oospores of *P. effusa* may be viable for several years in the environment despite adverse conditions^6^. Recently, oospore infestations were reported in about 19% of commercial spinach seed lots^10^. The movement of pathogen through infested seeds as oospores introduces novel recombinant isolates into spinach production sites and can spread the disease rapidly over large geographical regions^9^. Additionally, *P. effusa* produces tens of thousands of asexual spores per plant, which disperse aerially and initiate secondary infections within a field, or primary infections at another site, in distant fields^9,11^.

Increasing numbers of races, or new virulent pathotypes of *P. effusa*, have been detected using a set of differential spinach cultivars and reported in recent years in the US and other countries^12,13^. The emergence of virulent isolates capable of overcoming resistance genes is more common in pathogens such as downy mildews since they produce many asexual spores and retain high recombination rates^14,15^, but their emergence is exacerbated by sexual reproduction, as noted by the presence of oospores in the population^10^. Research efforts on *P. effusa* have focused on race phenotyping and understanding the basic epidemiology of the disease^9^. However, the recent public release of the genome sequences of *P. effusa* isolates of races 12, 13, and 14 have provided additional tools for genetic analysis of the pathogen^16,17^.

With the availability and affordability of next generation sequencing technology, transcriptome analyses between compatible (host susceptible) and incompatible (host resistant) plant-pathogen interactions have been useful to understand the molecular mechanisms that underlie the outcomes of these interactions in various pathosystems^18–23^. In plants, extracellular receptors serve as gatekeepers and recognize incoming pathogen-associated molecular patterns (PAMPs)^24^. Detection of PAMPs activates a basal defense system called pattern-triggered immunity (PTI)^24–26^. PTI can confer broad spectrum resistance through a basal defense network but pathogens coevolve with their host plants and can often breach the basal defense barrier by secreting effectors that are transported into the host cells^24,27^. Plant resistance (R) genes confer resistance against particular strains or races of a pathogen based on the recognition of specific effector molecules^24,27^. Plant R genes have been utilized as resources to develop resistant crop cultivars for several plant pathogens. In spinach, R genes at the RPF1 locus mediated resistance have been reported but functional roles of these genes conferring resistance to different races of *P. effusa* have not been thoroughly evaluated and validated^28^.

In this study, we performed genome-wide expression profiling of resistant and susceptible spinach-*P. effusa* interactions to investigate the defense mechanisms that confer resistance against *P. effusa* infection. For this purpose, we used resistant and susceptible spinach cultivars Solomon (also known as Lion) and Viroflay, respectively, which were inoculated with *P. effusa* and monitored for differential gene expression at two different time points, at an early stage of pathogen establishment and at the late stage of symptom expression and sporulation. We assembled a high-quality spinach reference genome sequence with annotation to facilitate this current analysis. In-depth analysis of genes differentially expressed during infection or in response to *P. effusa* yielded insights into the genetic basis of resistance and susceptibility in spinach and expression profiling of putative effector genes in *P. effusa* that are required for virulence and proliferation.

## Materials and methods

### Plant materials, pathogen, and pathogen inoculation

**Figure 1:**
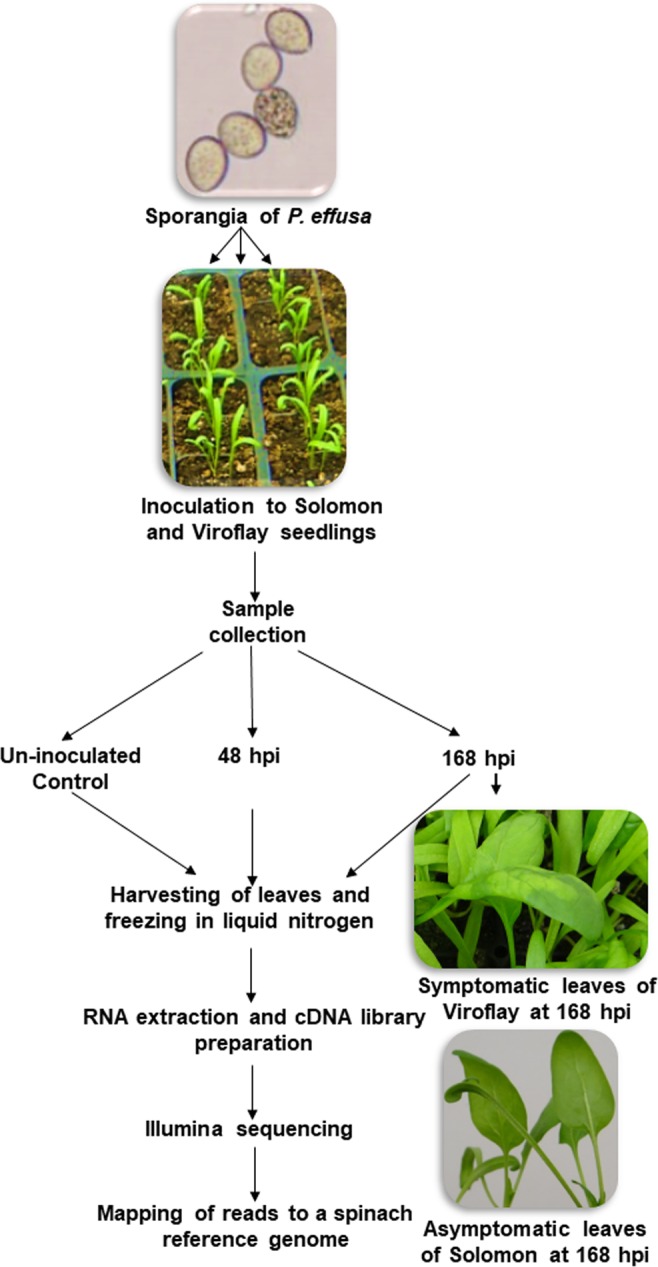
Experimental design and sample collection for RNA sequencing analyses of resistant and susceptible spinach cultivars in response to the spinach downy mildew pathogen, *Peronospora effusa*. Two-week-old spinach seedlings of the differential cultivars were inoculated with *P. effusa* sporangial suspensions. Leaves of the downy mildew resistant cultivar Solomon and susceptible cultivar Viroflay were collected at 48 and 168 hpi, and immediately transferred in liquid nitrogen. Un-inoculated control seedlings were also incubated overnight in the dew chamber, prior to collecting leaf samples. Three leaves per plant per treatment were pooled for total RNA extraction. cDNA libraries were prepared using three separate biological replicates per treatment.

Seeds of spinach differentials (Viroflay and Solomon) for detection of distinct pathotypes of *P. effusa* were sown in Sunshine potting mix (Sun Gro Horticulture) and allowed to grow for two weeks in the glasshouse under ambient conditions^12^. Viroflay is a semi-smooth leaf cultivar and susceptible to all known races of *P. effusa* while Solomon is a smooth leaf cultivar resistant to race numbers 1–9 and 11–16^29^ Two-week old seedlings were spray-inoculated with freshly harvested sporangia of *P. effusa* with the concentration of nearly 1 ×10^4^ sporangia/ml or remained un-inoculated as control plants. (Fig. 1). All seedlings were incubated in the dark for 24 hours in a dew chamber under high humidity (> 90%) at 18 ^o^C. The seedlings were moved into a mist chamber maintained at 19 ^o^C fixed with water misters to provide high humidity. Both *P. effusa* inoculated and un-inoculated control plants were remained in the mist chamber under a 12:12-h light-dark photoperiod for five days and were returned to the dew chamber for 24 hours in the dark at 18 ^o^C to stimulate sporulation^12^.

### Sample collections and experimental design

The experimental design is outlined in Fig. 1. Briefly, pathogen-inoculated leaf samples from cultivars Viroflay and Solomon were each collected at 48, and at 168 hours post-inoculation period (hpi). The un-inoculated control leaf samples were collected after 24-hr incubation in the dew chamber. There were three biological replicates (leaves harvested from three individual plants) per treatment (Fig. 1). Three true leaves were harvested manually from each plant at the respective time points, transferred immediately into liquid nitrogen, and stored at −80 ^o^C until further processing.

### RNA extraction, library preparation, and sequencing

Total RNA was extracted from frozen leaf samples using the RNeasy Mini Kit (Qiagen, Valencia, CA) according to manufacturer’s instructions on 100 mg of tissue for each sample, and yields were quantified using a Qubit 2.0 Fluorimeter (Invitrogen, Carlsbad, CA). cDNA libraries were constructed following the protocol as described earlier by Zhong *et al*.^30^. Libraries were sequenced on an Illumina HiSeq 4000 (Illumina, San Diego, CA) at the DNA Technologies and Expression Analysis Core Laboratory, University of California, Davis to yield 364,359,078 total sequence reads (single-end 50 bp) from all samples (Supplementary Table 1).

### Reference genomes used in this study

The reference genome of *P. effusa* used in this study was derived from a race 13 isolate from Monterey County, California (National Center for Biotechnology Information accession: QKXF00000000.1). The sequencing and annotation of 8607 genes in this *P. effusa* genome is described in detail by Fletcher *et al*.^16^.

The reference spinach genome of cultivar Viroflay used in this study was sequenced using Pacific Biosciences (Menlo Park, CA) technology to 70X coverage and assembled *de novo* using a hierarchical genome-assembly process and Celera assembler software^31^. The genome sequence, 890,266,198 bp in length, was annotated by Beijing Genomics Institute (Beijing, China) using MAKER^32^ and yielded a total of 34,878 genes in this spinach reference genome.

### Mapping sequence reads to the reference genomes and identification of differentially expressed genes

The mRNA-sequence reads obtained from each of the experimental spinach leaf samples were trimmed to remove adapter sequences using CLC Genomics Workbench 11.0.1 (http://www.clcbio.com). Trimmed reads were mapped to the spinach and *P. effusa* reference genomes using the default setting of RNA-Seq analysis tool in CLC Genomics. To ascertain the expression values, gene and mRNA tracks were created with CLC using both spinach and *P. effusa* genomes and their respective generic feature format files. Total gene reads, or in other words the total number of reads mapped to genes in the reference genome, were used to assess differential expression (Supplementary Table 1). An equally mapped read to both genic and intergenic regions was also considered as a mapped gene and included in the total counts. Reads that mapped outside of the annotated genes were counted as intergenic hits only and were excluded from the total gene reads. A two-dimensional heatmap was developed by clustering the expression values of individual genes across the samples at 48 and 168 hpi, respectively. The normalized log count per million (CPM) was calculated for each gene as an expression value. The Manhattan distance was used to calculate pairwise distances between all clusters where two closest clusters were merged into a single new cluster^33^. Furthermore, a three-dimensional principal component analysis (PCA) was performed using the default setting in CLC Genomics where normalized log CPM values were used as expression values. In the PCA analysis, PC1, PC2 and PC3 displayed the direction with the maximum, intermediate and minimum variability respectively, in the data.

In the RNA-seq tool in CLC Genomics Workbench (version 11.0.1; http://www.clcbio.com), we used the differential expression setting to determine the statistical significance of expressed genes in Solomon versus Viroflay at un-inoculated control and inoculated: 48 hpi and 168 hpi. We uploaded gene expression tracks of each samples containing total exon reads which were chosen to calculate the fold change and p-value statistics for the individual gene. Subsequently, we selected the metadata file containing all information including sample name, genotype (Solomon/Viroflay), time point of sample collection, and lane used while sequencing. For the experimental design and comparisons, we selected ‘genotype’ to test the differential expression, ‘lane’ for the control factor, and ‘all group pairs’ for the comparison. The analysis was conducted separately for each time point. Each expressed gene was modeled by using a generalized linear model (GLM)^34^, and calculated the dispersion estimate by a linear combination of the probability for that particular gene and its neighboring genes with similar average expression amounts. The GLM assumes the negative binomial distribution of the expression data. In the negative binomial distribution, dispersion of expression values serves as a free parameter indicating the homogeneity or heterogeneity i.e. smaller or greater variation in expression levels for a particular gene. We used Wald test^34^ for statistical testing and identified the significantly expressed genes at 48 hpi and 168 hpi between cultivars Solomon and Viroflay. The Wald test provided the p-value statistics of fold change of each gene between treatments by testing a non-zero coefficient. Species distribution of all BLAST^35^ hits of spinach genes was performed using the default setting of Blast2GO 5 PRO (https://www.blast2go.com/) program.

### Functional and pathway enrichment analysis of differentially expressed genes

Functional annotations of 34,878 spinach genes were generated through BLAST searches using CloudBlast in Blast2GO 5 Pro (https://www.blast2go.com/) program. The Blasted sequences were then mapped, annotated, and assigned the gene ontology (GO) IDs and functional description. The Fisher’s Exact Test was used in Blast2GO to perform a GO enrichment analysis to identify enrichments of genes involved in incompatible *P. effusa*-spinach interactions. Differentially expressed genes with a False Discovery Rate (FDR) threshold of 0.1% and a fold change> 8.0 were used for GO enrichment analysis. The Enzyme Code and KEGG (Kyoto Encyclopedia of Genes and Genomes; Kanehisa Laboratories)^36–38^ analysis was performed using KEGG maps to find the biochemical pathways that are regulated by significantly expressed genes in the different treatments.

### Single nucleotide polymorphism calling

The occurrence of single nucleotide polymorphisms (SNPs) in the expressed genes may modify the functional signature of these genes^39,40^. Therefore, we assessed SNPs in differentially expressed genes (FDR p-value = <0.01 from cultivars Solomon and Viroflay with minimum absolute fold change = 4) in spinach cultivars Solomon and Viroflay at 48 and 168 hpi, respectively. The variant detection toolbox in CLC Genomics Workbench 12 (http://www.clcbio.com) was used where the required variant probability was set to 90% with ploidy set to 2.

## Results

### Summary of transcript sequencing and mapping of sequence reads to the reference genome

A total of 364,359,078 sequence reads were generated from all samples included in this study using an Illumina sequencing platform (Supplementary Table 1). More than 95% reads were mapped to at least one position in the spinach reference genome for the majority of the samples, except for the 2^nd^ and 3^rd^ replications of cultivar Viroflay at 168 hpi, due to increased presence of pathogen transcripts sequenced in those samples. Furthermore, nearly 70% of total mapped reads from each sample were mapped to annotated genes within the spinach genome. Total gene reads include reads that span partly or entirely within an intron, an exon or an exon-exon junction, reads mapping fully within an exon, and reads that mapped to an exon junction. All other reads mapped partly or entirely between genes and were reported as mapped to the intergenic region. About 30% of total mapped reads from all samples were mapped to the intergenic regions in the genome.

**Figure 2:**
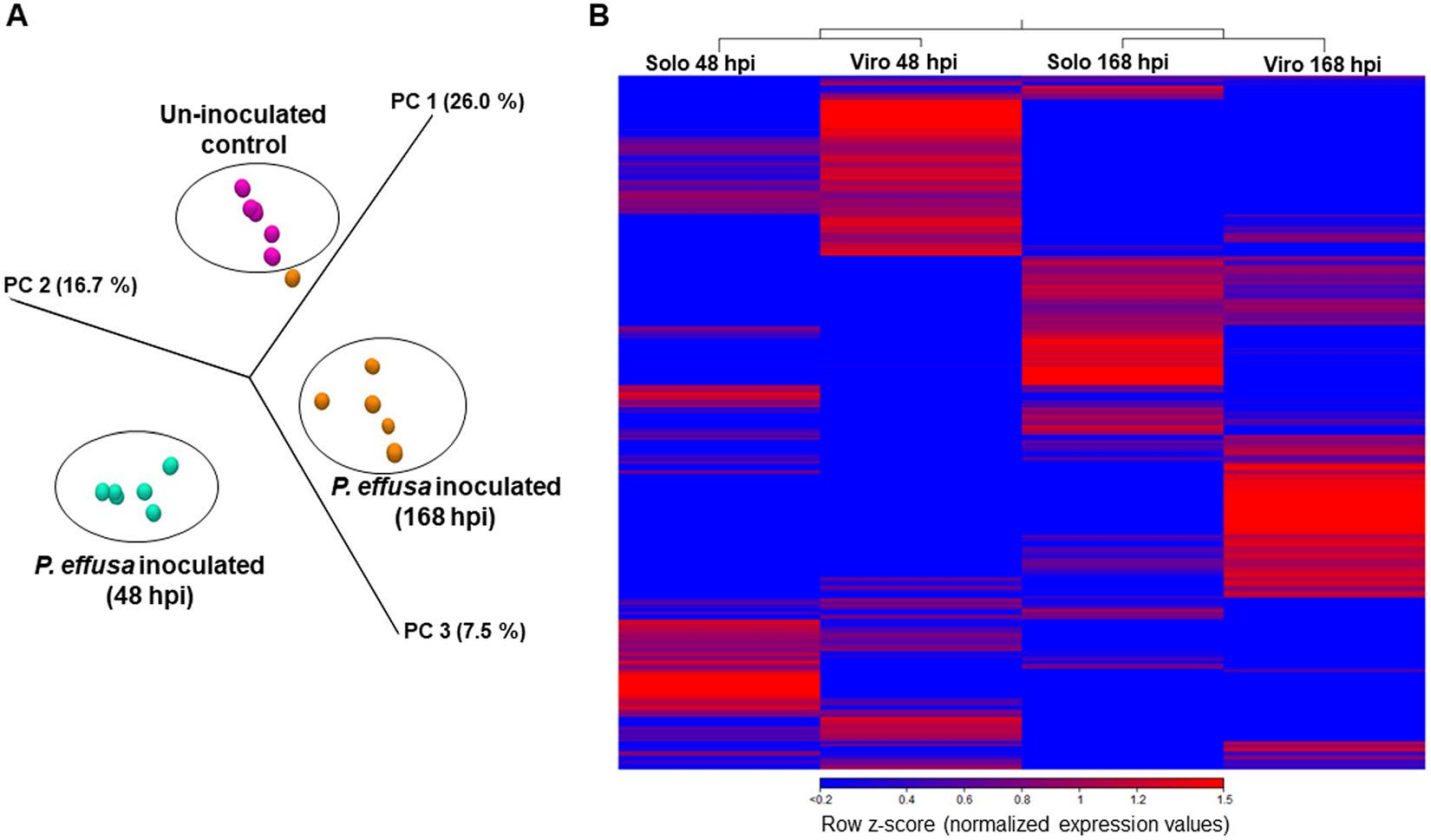
RNA-sequencing analyses of gene expression patterns in resistant and susceptible cultivars Solomon and Viroflay, respectively, inoculated with *Peronospora effusa*, or in the un-inoculated control treatment. (**A**) The principal component analysis of expression values across samples from cultivars Solomon and Viroflay in response to infection. (**B**) Heat map of expression values clustered in Solomon (Solo) and Viroflay (Viro) at 48 hours post-inoculation period (hpi) and 168 hpi, respectively. The expression value refers to normalized log counts per million, calculated for each gene.

The PCA showed clustering of expression values according to treatments i.e. un-inoculated control, *P. effusa*-inoculated 48 hpi, and *P. effusa*-inoculated 168 hpi, respectively (Fig. 2A). PC1, PC2, and PC3 specified varied gene counts from 26%, 16.7%, and 7.5%, respectively, from the replicated samples. A heatmap of hierarchical clustering showed that most genes expressed at higher levels in cultivar Solomon were expressed at lower levels in cultivar Viroflay and vice-versa, at 48 hpi and 168 hpi (Fig. 2B), clearly reflecting changes in metabolism and defense-associated gene expression during a late stage of pathogen colonization at 168 hpi, when leaf symptoms were present.

### Homologous species distribution

The reference spinach genome from cultivar Viroflay used in our analysis was 890,266,198 bp long and comprises of 34,878 genes (see materials and methods). The output received from the BLAST of the spinach gene set against the National Center Biotechnology Information database was used to develop the species distribution as determined by BLAST hits to validate transcriptome sequence identity (Supplementary Fig. 1). As anticipated, the maximum number of BLAST hits was associated primarily with *Spinacia oleracea* (84,705), followed by those of the two closely related species of *Chenopodium quinoa* (45, 614), and then *Beta vulgaris* subsp. *vulgaris* (29,727) (Supplementary Fig. 1).

### Detection of single nucleotide polymorphisms in expressed genes in cultivars Solomon and Viroflay

In our experiments, we detected SNPs in differentially expressed genes at a frequency of 0.0003% and 0.0006%, at 48 hpi and 0.002% and 0.006% at 168 hpi in spinach cultivars Solomon and Viroflay, respectively. At 48 hpi, the frequencies of 0.5% to 0.3% SNPs and 0.4% to 0.1% SNPs at 168 hpi in cultivars Solomon and Viroflay, respectively, detected in the same locations in the genome. Though SNPs in expressed genes may modify their functional signatures^39,40^, the extent of SNPs in significantly differentially expressed genes in cultivars Solomon and Viroflay was negligible. The SNP frequencies detected in our studies were not anticipated to have significant effects on the expression profiles of the genes or their functions.

### Overview of differential expression

**Figure 3:**
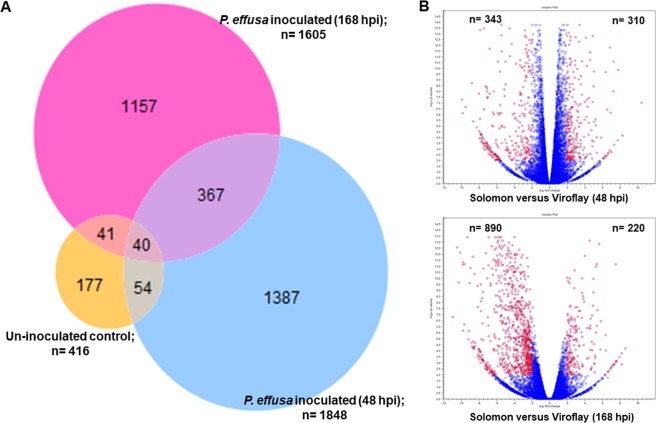
Venn diagram and Volcano plots of differentially expressed genes (DEGs), as determined by RNA-sequencing analysis in spinach cultivars Solomon (*Peronospora effusa*-resistant) and Viroflay (*P. effusa*-susceptible) at different time points after inoculation with the spinach downy mildew pathogen, *P. effusa*. (**A**) The Venn diagram displays significant differential gene expression values (FDR p-value = <0.01) with the minimum absolute fold change = 2. (**B**) Volcano plots of DEGs at 48 hours post-inoculation period (hpi) and 168 hpi, red circles indicate significant DEGs (FDR p-value = <0.01) with minimum absolute fold change = 4.

To determine the host response in resistant cultivar Solomon and susceptible cultivar Viroflay to *P. effusa* infection, differentially expressed genes were examined at an early stage of infection 48 hpi and at the commencement of disease symptoms and sporulation of pathogen at 168 hpi. At 168 hpi, no disease symptoms were observed in cultivar Solomon, but chlorosis together with sporulation was observed on the upper surface and underside of leaves, respectively, in cultivar Viroflay. A total of 23,063 and 23,364 genes were differentially expressed (Fold change = > ±1.0) at 48 and 168 hpi in cultivars Solomon and Viroflay, respectively (Supplementary File 1). However, only 416, 1,848, and 1,605 genes were expressed significantly (FDR p-value = <0.01 and minimum absolute fold change = 2) in accordance with the infection process (Fig. 3A; Venn diagram). A higher number of genes, 1387, was expressed uniquely at an early infection establishment stage at 48 hpi versus 1157 at 168 hpi. At 48 hpi, 310 genes were significantly up-regulated in Solomon versus Viroflay while 343 genes were down-regulated in Solomon versus Viroflay (Fig. 3B) (FDR p-value = <0.01 and minimum absolute fold change = 4). There was a marked shift in the proportions of up- and down-regulated genes at 168 hpi, reflecting the fact that Viroflay tissue was colonized and symptomatic at 168 hpi. At 168 hpi, 220 genes were significantly up-regulated while 890 genes were down-regulated (FDR p-value = <0.01 and minimum absolute fold change = 4) in Solomon versus Viroflay (Fig. 3B).

Among significantly expressed genes at 48 and 168 hpi in cultivar Solomon versus Viroflay, 85 were up-regulated and 112 were down-regulated at both time points while 214 genes that were up-regulated and 227 that were down-regulated were only expressed at 48 hpi. Similarly, there were 131 up-regulated and 767 down-regulated genes at 168 hpi in cultivar Solomon (Table 1). The list of significantly expressed genes at both 48 and 168 hpi time points or at either time point and their putative functions are presented in Supplementary File 2. To investigate the overall gene expression of the *P. effusa-*resistant cultivar Solomon and compare these values with the *P. effusa*-susceptible cultivar Viroflay, we combined expression values of each time point, and for each treatment of cultivar Solomon, and compared these individually with expression values in susceptible cultivar Viroflay at 48 hpi and 168 hpi, respectively. The up- or down-regulated expression patterns of the majority of the genes were similar to our results showing pairwise comparisons between the two cultivars at each time point.

**Table 1:** Summary of DEGs in *Peronospora-effusa* resistant cultivar Solomon versus susceptible Viroflay (FDR p-value = <0.01; minimum absolute fold change = 4) at 48 hours post-inoculation period (hpi) and 168 hpi.

Patterns of DEGs at 48 hpi versus 168 hpi	# DEGs	
	Up-regulated	Down-regulated
Only expressed at 48 hpi	214	227
Only expressed at 168 hpi	131	767
Expressed at both 48 and 168 hpi	85	112
•Up-reg. at 48 hpi but down-reg. at 168 hpi	11	
•Down-reg. at 48 hpi but up-reg. at 168 hpi	4	
Total expressed genes at 48 hpi	310	343
Total expressed genes at 168 hpi	220	890

### Differential expression of defense-associated genes

Plants have evolved specialized defense mechanisms which can recognize and discriminate between different environmental stresses or even different biotic threats^24^. Plant pathogens trigger up-regulation of defense genes, which may produce products that are directly antimicrobial or stimulate the biochemical pathways capable of producing antimicrobial metabolites^26,40^. Up-regulated genes significantly expressed at both time points, 48 and 168 hpi, may have roles in plant immunity, including the receptor-like protein kinase FERONIA, leucine-rich repeat (LRR) receptor-like serine/threonine-protein kinase, serine/threonine-protein kinase TIO (Supplementary File 2). Additionally, several genes encoding for transcription factor basic-helix-loop-helix (bHLH)-like or proteins with unknown function were also significantly up-regulated across both time points.

Furthermore, potential defense-related up-regulated genes expressed only at 48 hpi included homologs of the serine/threonine-protein kinase EDR10, LRR receptor-like serine/threonine-protein kinase, receptor-like serine/threonine-protein kinase ALE2, LRR receptor-like serine/threonine-protein kinase FLS2, and defensin-like protein. Similarly, up-regulated genes expressed uniquely at 168 hpi and probably crucial for the defense during late stages of downy mildew infection were ankyrin repeat-containing protein, 1-deoxy-D-xylulose-5-phosphate synthase, and defensin-like protein AX1. Six genes associated with early response to the pathogen were significantly expressed across both time points; however, these were induced at 48 hpi and repressed later at 168 hpi (Table 2). We also assessed the expression profile of common sets of genes relating to plant immunity such as protein kinases, pathogenesis related (PR) proteins, WRKY transcription factors, and ethylene- esponsive transcription factors or ethylene synthesizing enzymes. When compared to the fold change differences in expression values of resistant cultivar Solomon versus susceptible cultivar Viroflay, most of these genes were up-regulated at 48 hpi but down-regulated at 168 hpi (Supplementary file 3). Specifically, genes encoding for 1-aminocyclopropane-1-carboxylic acid (ACC) oxidase and ethylene transcription factors were significantly induced at 48 hpi but only a few of these genes were induced at 168 hpi. Two terminal enzymes in the phenylpropanoid pathway are chalcone synthase and cinnamoyl-CoA reductase, which are responsible for biosynthesis of flavonoids and lignin, respectively, in plants^41^. Genes encoding for cinnamoyl-CoA reductase 1-like were induced at 48 hpi but both cinnamoyl-CoA reductase and chalcone synthase-like genes were highly repressed at 168 hpi (Table 3).

**Table 2:** List of significant DEGs (Solomon versus Viroflay; FDR p-value = <0.01) up-regulated at 48 hours post-inoculation period (hpi) but down-regulated at 168 hpi with *Peronospora effusa* and their putative roles in host resistance/predicted function, nonsignificant values are not shown and listed as hyphens.

Gene identifier	Description	Fold change	
		48 hpi	168 hpi
Spiol01Chr13941	farnesol kinase, chloroplastic-like	13.36	−4.45
Spiol06Chr21962	probable WRKY transcription factor 41	10.39	−4.67
Spiol02Chr30362	probable WRKY transcription factor 41	5.63	−6.37
Spiol01Chr14208	uncharacterized protein LOC110804510	4.31	−6.6
Spiol05Chr34466	probably inactive leucine-rich repeat receptor-like protein kinase At5g48380	5.16	−7.03
Spiol04Chr12186	G-type lectin S-receptor-like serine/threonine-protein kinase RLK1	5.96	−9.15
Spiol04Chr11411	pathogenesis-related protein PR-1 type-like	3.17	−2.5
Spiol02Chr30613	pathogenesis-related protein PR-1 type-like	—	−24.55
Spiol06Chr22289	glutathione S-transferase-like	5.25	−103.89

**Table 3:** List of phenylpropanoid pathway-associated differentially expressed genes in resistant cultivar Solomon versus susceptible Viroflay (FDR p-value = <0.01) that were up-regulated at 48 hours post-inoculation period (hpi) but down-regulated at 168 hpi, nonsignificant values are not shown and listed as hyphens.

Gene identifier	Description	Fold change	
		48 hpi	168 hpi
Spiol04Chr09667	cinnamoyl-CoA reductase 1-like	2.94	—
Spiol06Chr22561	cinnamoyl-CoA reductase 1-like	2.12	—
Spiol04Chr09666	cinnamoyl-CoA reductase 1-like	—	−48.80
Spiol04Chr09668	cinnamoyl-CoA reductase 1-like	—	−5.68
Spiol06Chr22272	cinnamoyl-CoA reductase 1-like	—	−2.58
Spiol01Chr13283	chalcone synthase-like	—	−65.17
Spiol01Chr14613	chalcone synthase 2-like	—	−40.90
Spiol02Chr28595	chalcone synthase-like	—	−28.90

**Figure 4:**
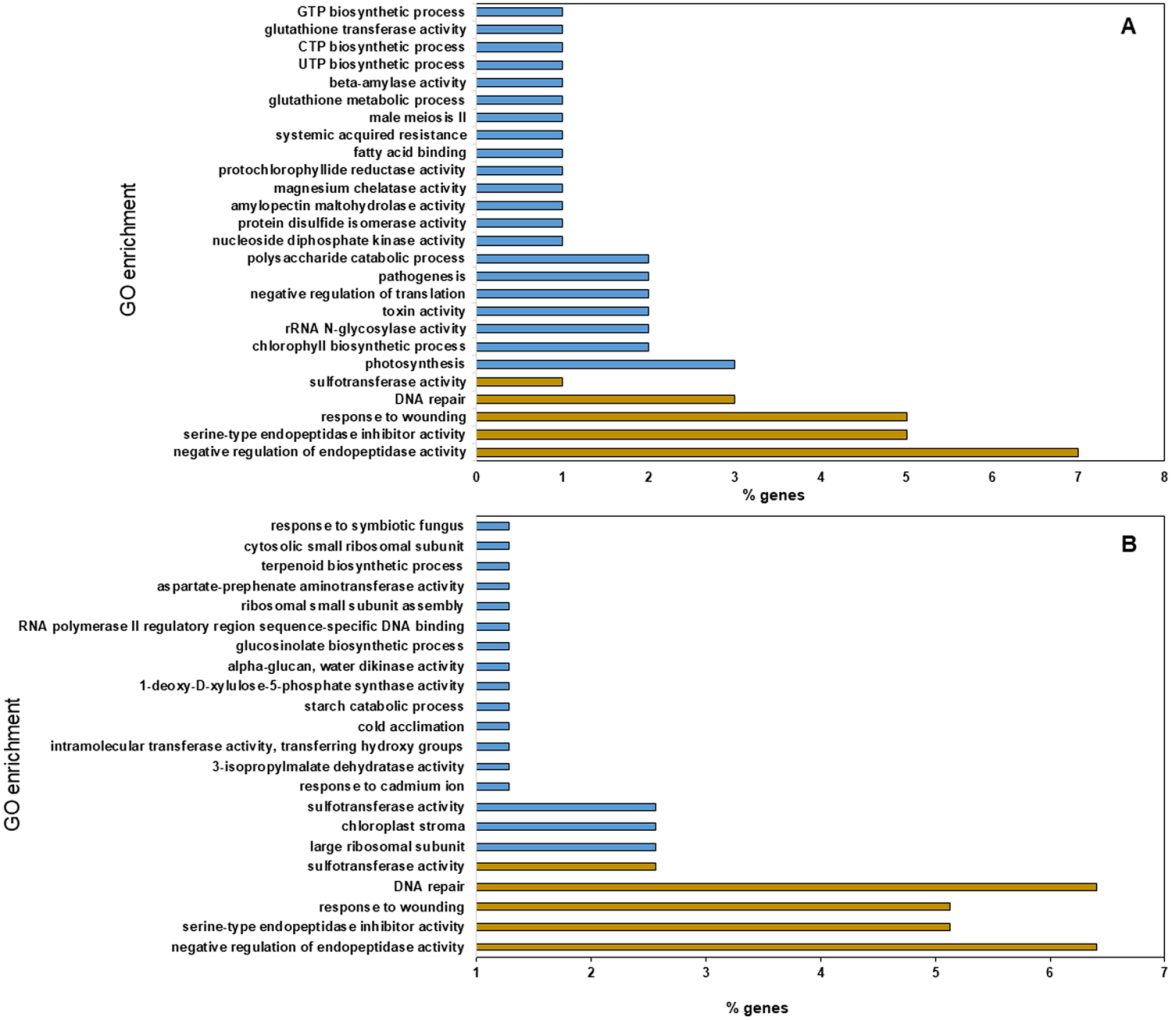
Gene ontology (GO) term enrichment derived from *Peronospora effusa-*spinach interactions, in which both resistant and susceptible cultivars were compared. (**A**) GO term enrichment at 48 hours post-inoculation period (hpi), and (**B**) GO term enrichment at 168 hpi resistant cultivar Solomon versus susceptible Viroflay. GO term enrichment was computed using significantly up-regulated DEGs at 48 and 168 hpi, respectively. Histograms with gold color indicate GO terms enriched across both time points.

A total of 100 and 78 significantly up-regulated genes (FDR p-value = <0.001 and Fold change = > 8.00) at 48 and 168 hpi, respectively, were used to assign GO terms and summarize their functional description. At 48 hpi, 155 GO terms were assigned, of which 60 were associated with biological process, 34 cellular component, and 61 molecular function. At 168 hpi, 126 GO terms were assigned, of which 45 were assigned to the biological process, 27 cellular component, and 54 molecular function (Supplementary File 4). GO enrichment analysis was performed using up-regulated genes at 48 and 168 hpi. At 48 hpi, specific GO terms were associated with up-regulated genes that may have roles in defense responses, wounding, toxin activity, pathogenesis, systemic acquired resistance, glutathione transferase activity, etc. (Fig. 4A). At 168 hpi, significantly enriched GO terms associated with host defense responses at late stages of infection were terpenoid biosynthetic process, alpha-glucan, water dikinase activity, response to wounding etc. (Fig. 4B). The GO terms including negative regulation of endopeptidase activity, serine-type endopeptidase inhibitor activity, response to wounding, DNA repair, and sulfotransferase activity were enriched at both the 48 and 168 hpi time points.

KEGG pathway analysis was performed using genes significantly up- and down-regulated (FDR p-value = <0.001 and minimum absolute fold change = 8.00) at 48 and 168 hpi, respectively. The pathway analysis identified 13 and 5 up-regulated genes involved in 14 metabolic pathways at 48 and 168 hpi, respectively. Likewise, 21 and 91 down-regulated genes in 33 metabolic pathways, at 48 and 168 hpi, respectively (Supplementary Table 2). At both 48 and 168 hpi, but particularly at 168 hpi, there were multiple down-regulated genes that were identified as involved in thiamine metabolism and phenylpropanoid pathways.

### Expression profiling of the *P. effusa* transcriptome during incompatible and compatible interactions

**Figure 5:**
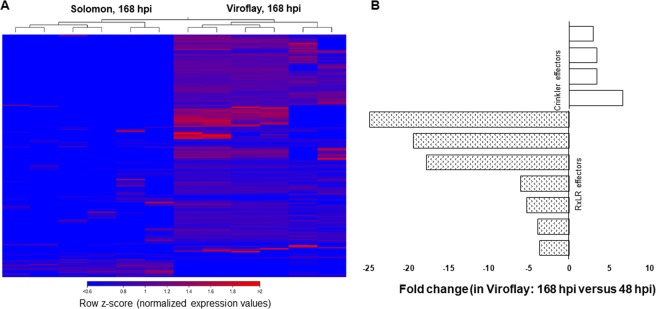
Expression profile and differential expression of *Peronospora effusa* transcriptomes/translated proteins during incompatible and compatible interactions with resistant spinach cultivar Solomon and susceptible cultivar Viroflay, respectively. (**A**) Heat map of expression values clustered in resistant cultivar Solomon and susceptible cultivar Viroflay at 168 hours post-inoculation period (hpi). (**B**) Fold change of putative *P. effusa* effectors (168 hpi versus 48 hpi) in response to infection in susceptible cultivar Viroflay.

The RNA-seq reads mapped to the genome of *P. effusa* race 13 were used primarily to examine the temporal expression patterns of putative effector proteins, which may influence how the pathogen infects and colonizes spinach. At 48 hpi, less than 1% of total reads retrieved from susceptible cultivar Viroflay were mapped to the *P. effusa* genome, while at 168 hpi, nearly 25 to 48% of total reads were mapped to the *P. effusa* genome. This is in contrast to the resistant cultivar Solomon, where the mapping percentage was approximately the same at both 48 hpi and 168 hpi but less than 1% of total reads. In the Viroflay samples, several *P. effusa* genes were strongly expressed at 168 hpi but only a few genes were activated and weakly expressed in the samples derived from the cultivar Solomon (Heatmap; Fig. 5A). Differential expression of genes encoding homologs of RxLR and crinklers, two major effector protein families in oomycetes, was calculated in Viroflay using expression values at 168 and 48 hpi. Interestingly, only effector proteins of the crinkler family were significantly up-regulated (FDR p-value = <0.05) while RxLR effectors were significantly down-regulated when expression values were compared at 168 hpi versus 48 hpi (Fig. 5B). We performed BLASTX^35^ searches of sequences encoding these putative effectors and checked for homologs in the *Phytophthora infestants* T30–4 database^42^. These putative effector proteins from *P. effusa* race 13 shared a substantial homology with RxLR and crinkler proteins of *P. infestants* T30-4. The RxLR and crinkler effectors from *P. effusa* shared 30 to 82% homology with RxLR proteins from *P. infestans* and 50% homology with the identified Crinklers of *P. infestans*.

Additional pathogen genes of interest included those encoding peroxidase/catalases, peroxide reductases, and several NPP1 type necrosis inducing-like proteins that were identified at the late infection stage of 168 hpi (Supplementary File 5). Each of these types of proteins may contribute to host colonization since catalase-peroxidases are important for virulence in some plant pathogens by protecting against hydrogen peroxide produced in the interaction with the host^43^.

### Infectivity of *P. effusa* in susceptible cultivar Viroflay at 48 hpi and 168 hpi

Sporangia of *P. effusa* germinated overnight, producing germ tubes that can potentially invade the leaf surface (Supplementary Fig. 2A). The germ tube differentiated and grew rapidly, generating networks of infectious hyphae in the susceptible cultivar Viroflay (Supplementary Fig. 2B).

## Discussion

The recent advances in RNA sequencing have enabled unprecedented depth and accuracy for transcriptome analyses. Using this powerful approach, differential gene expression associated with resistant and susceptible crop genotypes has been examined in various pathosystems to understand the molecular basis of plant defense responses^19,44–47,48^. In this study, we applied this tool along with the newly available spinach and *P. effusa* genome resources, to analyze gene expression during incompatible and compatible spinach- *P. effusa* interactions. We used resistant and susceptible spinach cultivars Solomon and Viroflay, respectively, and inoculated with *P. effusa* and monitored the temporal pattern of gene expression at 48 and 168 hpi, respectively. We examined genes expressed in both host (spinach cultivars) and pathogen (*P. effusa*) sides during host-microbe interactions, at 48 and 168 hpi.

Diverse and distinct spinach gene sets were expressed during incompatible interactions between *P. effusa* and cultivar Solomon versus compatible interactions between *P. effusa* and cultivar Viroflay. Genes significantly up-regulated at 48 and 168 hpi in cultivar Solomon were of particular interest since these were hypothesized as critical for resistance to pathogen invasion. Plant immunity, which may occur during an incompatible interaction, operates at two levels: a basal immunity that is regulated through pattern recognition receptors upon perception of pathogen-associated molecular patterns and a race or cultivar-specific resistance mediated through resistance genes that recognize and inactivate pathogen-derived effector molecules^26,41^. In spinach, race-specific resistance has been reported to the downy mildew but the functional mechanisms of host resistance remain undetermined^28^. During incompatible plant-pathogen interactions, plant R proteins recognize and inactivate pathogen-derived effector molecules^26,41^, which often leads to the hypersensitive cell death at the site of pathogen attack^27,49^. The hypersensitive response is often associated with extensive transcriptional reprogramming and activation of several defense genes to sustain the immunity^27,50^. Plant R genes may encode a combination of conserved domains, including LRRs, protein kinases, nucleotide binding site (NBS)s, and in at least one case, a WRKY transcription factor domain^47,51^. These domains confer the potential to perceive and signal inputs from external stimuli^27,49,52^. Furthermore, there is increasing evidence that receptor-like kinases regulate plant growth and development and also modulate defense signaling pathways by sensing modifications in plant cell wall architecture^53–56^. Also, phytohormones, mainly salicylic acid, ethylene and jasmonic acid mediated signaling networks, have been explored regarding their significance in the induction and maintenance of plant immunity^22^.

In our study, we found significant upregulation of genes encoding a zinc finger CCCH protein, ACC oxidase, receptor-like protein kinase FERONIA, LRR receptor-like serine/threonine-protein kinase, and serine/threonine-protein kinase TIO during early (48 hpi) and late (168 hpi) stages of infections in the resistant cultivar Solomon. The occurrence of zinc finger domains has been documented in many disease resistance genes that are required for host resistance during incompatible interactions^57^. The receptor-like kinase FERONIA is not only important for normal plant growth and reproduction, but also induces the liberation of reactive oxygen species in response to the changes in the cell wall^58,59^. We speculate that the encoded FERONIA homolog in spinach may be a first responder to *P. effusa* as sporangia germinate and initially invade through the cell walls in spinach leaves by direct penetration^9^. The instantaneous increase of reactive oxygen species at the infection site, possibly activated the enzyme glycosyltransferase, and involves the biosynthesis and strengthening of plant cell walls^60^. The upregulation of genes associated with glycosyltransferase activity has been reported upon inoculation of *Arabidopsis* plants with plant pathogens *Botrytis cinerea* and *P. infestans*^61^. The enriched GO term ‘response to wounding’ at both 48 and 168 hpi also suggested that preserving cell wall integrity is an important step to retain the resistance over *P. effusa* invasion.

The significant upregulation of genes associated with PR-1 protein, glutathione-S-transferase, phospholipid hydroperoxide glutathione peroxidase, peroxidase was observed particularly at 48 hpi which are reported to minimize the oxidative damage in plants in response to biotic and abiotic stresses^62,63^. Homologs of these gene products restrict the oxidative damage in host tissue and initiate several succeeding downstream signaling cascades^62,64^. The PR-1 gene has been thoroughly studied in the past for its role in defense to different pathogens, including *P. infestans*^65,66^. Additionally, genes encoding R-proteins such as LRR receptor-like serine/threonine-protein kinase, ankyrin repeat-containing protein NPR4-like, G-type lectin S-receptor-like serine/threonine-protein kinase, and nucleoside diphosphate kinase 2 were highly expressed at 48 hpi in cultivar Solomon compared to Viroflay. It is possible that extracellular domains of these receptors physically interact with the *P. effusa* specific PAMP molecules and activate the downstream defense signals that are critical to subsequently suppress the infection process. Some of these genes may be targeted by inhibitory RNAs or CRISPR-Cas9 approaches to assess their functions during a resistant response.

Certain host genes, including those encoding transcription factors, were highly differentially expressed in this study, in susceptible and resistant spinach-*P. effusa* interactions. The members of the bHLH family are involved in cooperating, through various phytohormone-regulated signaling pathways to regulate plant immunity^67,68^. We found significant up-regulation of genes encoding transcription factor bHLH3-like proteins in the resistant interaction at both 48 and 168 hpi. Spinach homologs of those genes encoding plant defensin-like proteins were also strongly expressed during the incompatible interaction, indicating their involvement in defense in this interaction. A number of studies have shown the inhibitory effect of plant defensin peptides to various fungal and bacterial pathogens^69–72^. Genes encoding ACC oxidase homologs in spinach were up-regulated across the infection times in our experiments. ACC oxidase is involved in the biosynthesis of the plant hormone ethylene and the presence of ethylene subsequently activates the downstream genes which can inactive the effector proteins and restore the plant immunity^73,74^. The increased synthesis of ethylene has been reported as one of the earliest responses of plants to a wide variety of plant pathogens, including downy mildew pathogens^48,75,76^. A gene encoding for AP2-like ethylene-responsive transcription factor was also significantly up-regulated at 48 hpi and was reported to have an important role in conferring resistance to plant pathogens^77,78^. The expression of AP2 transcription factor is activated by plant hormones including ethylene, which induces pathogenesis-related genes and activates defense signaling pathways^74,79^.

The flavonoid and phenylpropanoid pathways play a central role in plant defense by providing structural barriers through lignification of cell walls and in establishing chemical barriers by activating defense genes involving in signaling networks for resistance to pathogen infection^80,81^. In the case of grape downy mildew, a thiamine-induced phenylpropanoid pathway was reported as a crucial mechanism to confer resistance to the *Plasmopara viticola* infection^82^. At 168 hpi, there was notable up-regulation in the resistant interaction of gene Spiol06Chr03650, encoding a probable 1-deoxy-D-xylulose-5-phosphate synthase (DXS). DXS catalyzes an early step in the isoprenoid biosynthetic pathway, which results in carotenoid and chlorophyll production in plants. In earlier studies, DXS transcripts were negatively correlated with late blight symptoms in potato, indicating its potential role in host defense against plant pathogens^83^. The gene encoding enzyme cinnamoyl-CoA reductase, a key enzyme in the lignin biosynthesis, was significantly up-regulated in the resistant cultivar Solomon at 48 hpi, which may have a role in lignification to confer tolerance to pathogen invasion. Conversely, at the 168 hpi, down-regulated genes associated with the phenlypropanoid biosynthesis pathway were highly expressed in the susceptible cultivar Viroflay versus cultivar Solomon. Down-regulation of key genes involved in phenlypropanoid biosynthesis may assist in pathogen proliferation. This down-regulation may be due to the delivery of pathogen effector proteins that target and suppress the expression of these host genes.

Examination of *P. effusa* revealed highly expressed genes encoding for RxLR and crinkler effectors, which were especially prevalent in the interaction with the susceptible cultivar Viroflay. A recent study revealed that the *P. effusa* genomes comprise various potential PAMP and effector molecules which can suppress the host immunity^16^. These effector proteins share significant sequence similarity with known effectors of *P. infestans*^42^. Our results suggested that RxLR effectors were actively expressed at the early stage of the infection while Crinkler effectors were active at the late stage of the infection. In this current study, RxLR effectors were associated with the vegetative invasive stage in the pathogen, likely facilitating host colonization and acquisition of nutrients from the host. The significant expression of RxLR effector genes was also reported at the early stage of infection (12 hpi) when susceptible potato plants were inoculated with virulent strains of *P. infestans*^84^. Thus, the expression of Crinkler effectors identified in this study at the late stage of infection may affect the host physiology to initiate the disease symptoms and the expansion of infected areas. Additional data may be mined from this data set generated in this study to further study mechanisms of infectious hyphal growth during spinach-*P. effusa* interactions. Though this awaits further validation, the peroxidase/catalases, peroxide reductases, and the NPP1 homolog expressed in the pathogen during late stages of infection may reflect the mounting effort on the part of the pathogen to combat high levels of certain reactive oxygen species produced by the plant, and also to inflict cell death at the late stage of infection when symptoms including both chlorosis and necrosis may be present. In *Arabidopsis* and parsley, NPP1 from *Phytophthora* can induce host defenses gene expression and the production of reactive oxygen species, and cause cell death^85,86^.

Mechanisms of downy mildew resistance in spinach were identified in this study, along with the candidate genes that may be useful in projects that aim to breed downy mildew resistance. Hypersensitive response-inducible genes such as PR-1 and glutathione-S-transferase and protein kinase FERONIA were strongly up-regulated in the resistant cultivar Solomon in response to *P. effusa*, and thus defense responses that occur via salicylic acid signaling and reactive oxygen species are likely similar to those described in other plant species^87–89^. Furthermore, multiple genes related to phenylpropanoid pathways were significantly down-regulated in the susceptible interaction and genes encoding ACC oxidase and AP2-like ethylene-responsive transcription factor were up-regulated in the resistant interaction. Thus, it is plausible that crosstalk between salicylic acid and ethylene signaling pathways and cell wall lignification undergirds the resistant interaction, but this requires further validation. In future studies, we will investigate the potential role of ethylene-based signaling in spinach cultivars, especially in relation to ethylene-modulated kinase signaling events conferring tolerance or resistance to pathogen invasion. This study and other studies that attempt to unravel the molecular basis of disease or resistance in the spinach-*P. effusa* interaction may enable development of effective management strategies for spinach downy mildew.

## Supplementary information

Supplementary Figure 1

Supplementary Figure 2

Supplementary File 1

Supplementary File 2

Supplementary File 3

Supplementary File 4

Supplementary File 5

Supplementary Table 1

Supplementary Table 2

## Data Availability

The RNA-seq data generated in this study have been deposited at National Center for Biotechnology Information under the BioProject identification number PRJNA603356. The reference spinach genome can be accessed through the repository at https://github.com/USDA-ARS-GBRU/Spinach_Peffusa.
