# Supplementary figures and images for: Transcriptional analyses of differential cultivars during resistant and susceptible interactions with Peronospora effusa, the causal agent of spinach downy mildew
Source: Sci Rep. 2020 Apr 21;10:6719. doi: 10.1038/s41598-020-63668-3 (PMC7174412; doi:10.1038/s41598-020-63668-3)

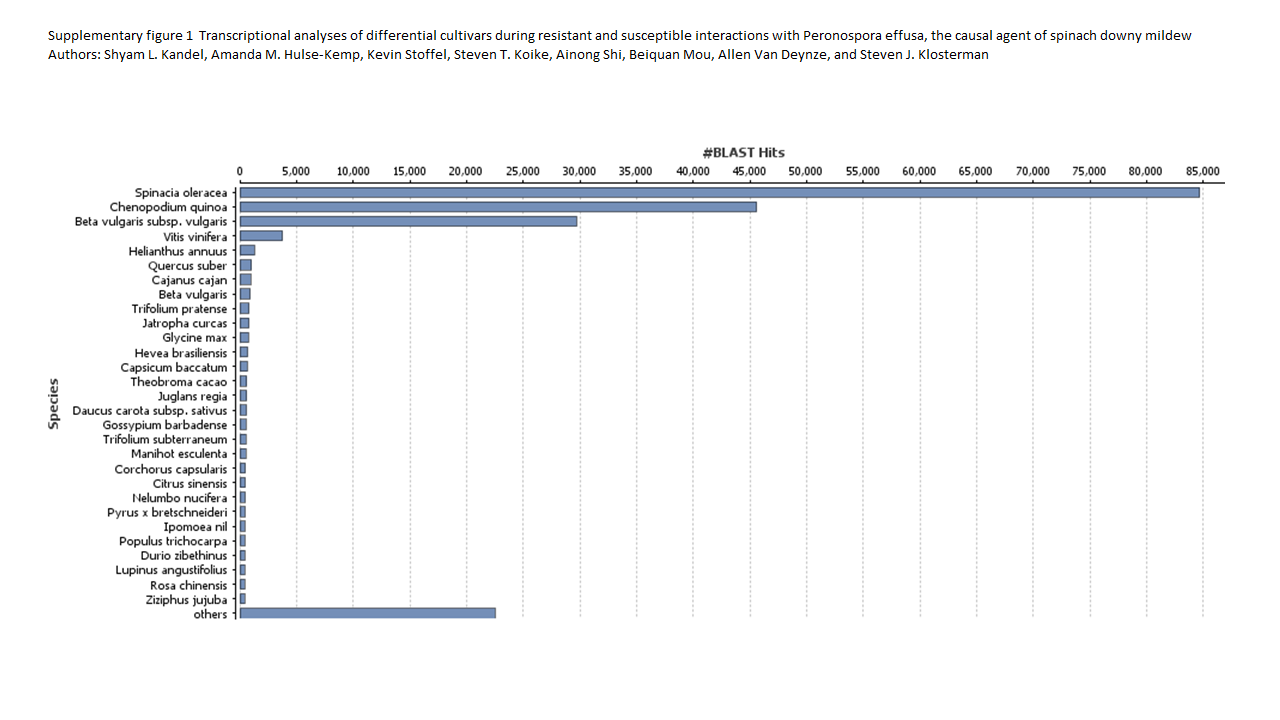

Supplement: Supplementary file 1 — Supplementary Figure 1 [file 41598_2020_63668_MOESM1_ESM.tif]

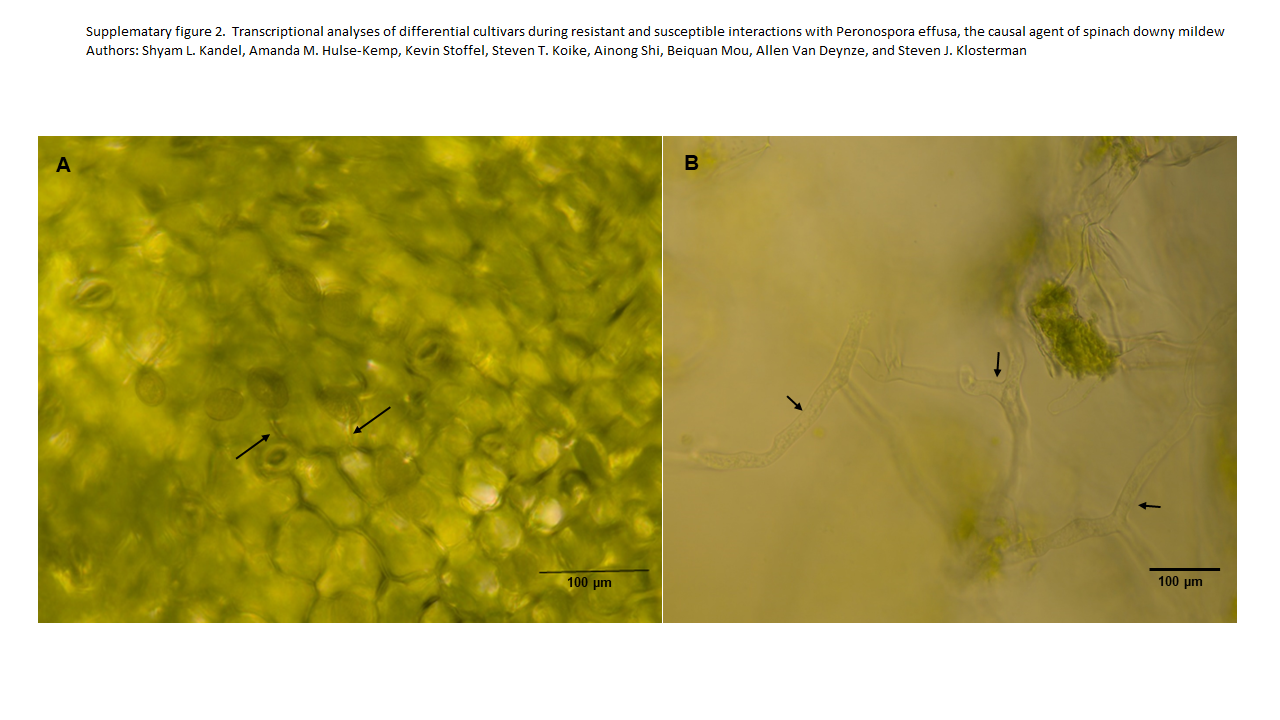

Supplement: Supplementary file 2 — Supplementary Figure 2 [file 41598_2020_63668_MOESM2_ESM.tif]
