# Supplementary material for: Transcriptional analyses of differential cultivars during resistant and susceptible interactions with Peronospora effusa, the causal agent of spinach downy mildew
Source: Sci Rep. 2020 Apr 21;10:6719. doi: 10.1038/s41598-020-63668-3 (PMC7174412; doi:10.1038/s41598-020-63668-3)
Supplement: Supplementary file 8 — Supplementary Table 1 [file 41598_2020_63668_MOESM8_ESM.docx]

**Transcriptional analyses of differential cultivars during resistant and susceptible interactions with *Peronospora effusa*, the causal agent of spinach downy mildew**

**Authors:** Shyam L. Kandel, Amanda M. Hulse-Kemp, Kevin Stoffel, Steven T. Koike, Ainong Shi, Beiquan Mou, Allen Van Deynze, and Steven J. Klosterman

Supplementary Table 1: Summary of RNA-seq reads and mapping of sequence reads to the reference genome.

| **Sample ID** | **Read counts^£^** | **Reads mapped %** | **Mapped to genes %** | **Mapped to intergenic %** |
| --- | --- | --- | --- | --- |
| L7-Solo-Un-inoculated Control-R1 | 11,571,266 | 97.82 | 70.42 | 29.58 |
| L8-Solo-Un-inoculated Control-R1 | 11,271,729 | 97.82 | 70.44 | 29.56 |
| L7-Solo-Un-inoculated Control-R2 | 10,797,967 | 97.34 | 70.37 | 29.63 |
| L8-Solo-Un-inoculated Control-R2 | 10513043 | 97.35 | 70.36 | 29.64 |
| L7-Solo-Un-inoculated Control-R3 | 10697872 | 97.51 | 70.33 | 29.67 |
| L8-Solo-Un-inoculated Control-R3 | 10439837 | 97.53 | 70.39 | 29.61 |
| L7-Solo-48 hpi-R1 | 11581582 | 97.88 | 70.72 | 29.28 |
| L8-Solo-48 hpi-R1 | 11299759 | 97.88 | 70.71 | 29.29 |
| L7-Solo-48 hpi-R2 | 11156234 | 97.53 | 70.37 | 29.63 |
| L8-Solo-48 hpi-R2 | 10980206 | 97.56 | 70.43 | 29.57 |
| L7-Solo-48 hpi-R3 | 11722688 | 97.74 | 70.3 | 29.7 |
| L8-Solo-48 hpi-R3 | 11393649 | 97.75 | 70.33 | 29.67 |
| L7-Solo-168 hpi-R1 | 10512063 | 97.65 | 69.84 | 30.16 |
| L8-Solo-168 hpi-R1 | 10343452 | 97.66 | 69.81 | 30.19 |
| L7-Solo-168 hpi-R2 | 9958853 | 97.46 | 67.31 | 32.69 |
| L8-Solo-168 hpi-R2 | 9825089 | 97.48 | 67.33 | 32.67 |
| L7-Solo-168 hpi-R3 | 10213593 | 97.36 | 69.84 | 30.16 |
| L8-Solo-168 hpi-R3 | 10179651 | 97.38 | 69.86 | 30.14 |
| L7-Viro-Un-inoculated Control-R1 | 9157584 | 97.05 | 70.91 | 29.09 |
| L8-Viro-Un-inoculated Control-R1 | 8996243 | 97.05 | 70.93 | 29.07 |
| L7-Viro-Un-inoculated Control-R2 | 10010944 | 97.55 | 70.5 | 29.5 |
| L8-Viro-Un-inoculated Control-R2 | 9949903 | 97.58 | 70.5 | 29.5 |
| L7-Viro-48 hpi-R1 | 10987282 | 96.94 | 70.06 | 29.94 |
| L8-Viro-48 hpi-R1 | 10828014 | 96.95 | 70.09 | 29.91 |
| L7-Viro-48 hpi-R2 | 10075672 | 92.96 | 70.86 | 29.14 |
| L8-Viro-48 hpi-R2 | 9931794 | 92.97 | 70.88 | 29.12 |
| L7-Viro-48 hpi-R3 | 10465715 | 96.94 | 70.96 | 29.04 |
| L8-Viro-48 hpi-R3 | 10179286 | 96.96 | 70.99 | 29.01 |
| L7-Viro-168 hpi-R1 | 12344217 | 96.47 | 69.76 | 30.24 |
| L8-Viro-168 hpi-R1 | 12008786 | 96.48 | 69.79 | 30.21 |
| L7-Viro-168 hpi-R2 | 10671766 | 48.28 | 68.5 | 31.5 |
| L8-Viro-168 hpi-R2 | 10512034 | 48.29 | 68.52 | 31.48 |
| L7-Viro-168 hpi-R3 | 12066831 | 73.16 | 68.32 | 31.68 |
| L8-Viro-168 hpi-R3 | 11714474 | 73.13 | 68.35 | 31.65 |

^£^Total amount of reads from individual sample was mapped to the spinach reference genome using CLC genomics Workbench 11.0.1. hpi: hours post inoculation, L: Lane used during sequencing, Solo: cultivar ‘Solomon’, Viro: cultivar ‘Viroflay’, and R: Replicate.
