# Supplementary material for: Transcriptional analyses of differential cultivars during resistant and susceptible interactions with Peronospora effusa, the causal agent of spinach downy mildew
Source: Sci Rep. 2020 Apr 21;10:6719. doi: 10.1038/s41598-020-63668-3 (PMC7174412; doi:10.1038/s41598-020-63668-3)
Supplement: Supplementary file 9 — Supplementary Table 2 [file 41598_2020_63668_MOESM9_ESM.docx]

**Transcriptional analyses of differential cultivars during resistant and susceptible interactions with *Peronospora effusa*, the causal agent of spinach downy mildew**

**Authors:** Shyam L. Kandel, Amanda M. Hulse-Kemp, Kevin Stoffel, Steven T. Koike, Ainong Shi, Beiquan Mou, Allen Van Deynze, and Steven J. Klosterman

Supplementary Table 2. KEGG pathway analyses of differentially expressed genes in spinach (resistant cultivar Solomon versus susceptible Viroflay) at 48 and 168 hours post inoculation with *Peronospora effusa.*

| **Pathway** | **Upregulated genes #** | | **Downregulated genes #** | |
| --- | --- | --- | --- | --- |
|  | **48 hpi** | **168 hpi** | **48 hpi** | **168 hpi** |
| Metabolism of xenobiotics by cytochrome P450 | 1 |  |  | 1 |
| Purine metabolism | 1 |  | 9 | 15 |
| Glutathione metabolism | 1 |  | 1 | 1 |
| Pentose and glucuronate interconversions | 1 |  | 1 | 1 |
| Starch and sucrose metabolism | 1 |  |  |  |
| Drug metabolism - other enzymes | 3 |  |  | 2 |
| Drug metabolism - cytochrome P450 | 1 |  |  | 1 |
| Porphyrin and chlorophyll metabolism | 2 |  |  |  |
| Pyrimidine metabolism | 1 | 1 |  |  |
| Biosynthesis of antibiotics | 1 |  | 1 | 3 |
| Valine, leucine and isoleucine biosynthesis | | 1 |  |  |
| Terpenoid backbone biosynthesis |  | 1 |  |  |
| Thiamine metabolism |  | 1 | 5 | 16 |
| Phenylalanine, tyrosine and tryptophan biosynthesis | | 1 |  |  |
| Phenylpropanoid biosynthesis |  |  | 1 | 11 |
| Pyruvate metabolism |  |  |  | 4 |
| Amino sugar and nucleotide sugar metabolism | |  |  | 4 |
| Nitrogen metabolism |  |  |  | 3 |
| Glycolysis / Gluconeogenesis |  |  |  | 2 |
| Oxidative phosphorylation |  |  | 1 | 2 |
| Cutin, suberine and wax biosynthesis |  |  |  | 2 |
| Drug metabolism - other enzymes |  |  |  | 2 |
| Carbon fixation in photosynthetic organisms | |  |  | 2 |
| Arginine biosynthesis |  |  | 1 | 2 |
| Alanine, aspartate and glutamate metabolism | |  |  | 2 |
| Citrate cycle (TCA cycle) |  |  |  | 2 |
| Metabolism of xenobiotics by cytochrome P450 | |  |  | 1 |
| One carbon pool by folate |  |  |  | 1 |
| Zeatin biosynthesis |  |  |  | 1 |
| Glyoxylate and dicarboxylate metabolism |  |  |  | 1 |
| Aminoacyl-tRNA biosynthesis |  |  |  | 1 |
| Butanoate metabolism |  |  |  | 1 |
| Riboflavin metabolism |  |  |  | 1 |
| Taurine and hypotaurine metabolism |  |  |  | 1 |
| Carbon fixation pathways in prokaryotes |  |  |  | 1 |
| beta-Alanine metabolism |  |  |  | 1 |
| Glycerolipid metabolism |  |  |  | 1 |
| Starch and sucrose metabolism |  |  |  | 1 |
| Cysteine and methionine metabolism |  |  | 1 | 1 |
